# Changes in the Serum Level of Vitamin D During Healing of Tibial and Femoral Shaft Fractures

**DOI:** 10.5812/traumamon.10946

**Published:** 2014-01-25

**Authors:** Hossein Ettehad, Ahmadreza Mirbolook, Fereshteh Mohammadi, Mohammadsadegh Mousavi, Hannan Ebrahimi, Ardeshir Shirangi

**Affiliations:** 1 Orthopedic Research Center, Poursina Hospital, Guilan University of Medical Sciences, Rasht, IR Iran

**Keywords:** Vitamin D, Fractures, Bone, Tibia, Femur, Cholecalciferol

## Abstract

**Background:**

Several systemic factors and hormones are thought to regulate the fracture healing process. Vitamin D has emerged as a compound or hormone that actively participates in the regulation of calcium homeostasis and bone metabolism.

**Objectives:**

The aim of this study is to determine the serum changes in the level of vitamin D during the acute healing period of tibial and femoral shaft fractures.

**Patients and Methods:**

This cross-sectional study included of 73 patients with tibial and femoral shaft fractures referred to the Poursina Hospital between February 2011 and February 2012. Changes in the serum levels of vitamin D were assessed three times in a period of three weeks (at the first visit, end of first week, and end of the third week). Variables such as age, gender, fractured bone, concomitant fracture of tibia and fibula, type of fracture, time of measurement and serum levels of 25-hydroxyvitamin D were assessed. All statistical analyses were performed using the SPSS software.

**Results:**

Forty tibial fractures and 33 femoral fractures were recorded. Mean vitamin D levels at the time of admission, after one week and at the end of the third week for the 73 participants included in the study were 39.23, 31.49, and 28.57 ng/mL, respectively. The overall reduction of vitamin D level was significantly more evident in the first week versus the following (P < 0.0001).

**Conclusions:**

Serum levels of vitamin D in patients with tibial or femoral fractures were reduced during the curative period of the fracture. This can be related the role of vitamin D in the formation and mineralization of the callus. Patients with tibial or femoral shaft fractures may benefit from the administration of vitamin D supplements during the fracture healing process.

## 1. Background

The healing of long bones after a fracture is a unique process that results in the restoration of normal bone anatomy and functionality after serious injuries (1). Several systemic factors and hormones are thought to regulate the fracture healing process. However, little is understood about how these hormones regulate local repair processes and control local cellular events (2). Vitamin D has emerged as a compound or hormone that actively participates in the regulation of calcium homeostasis and bone metabolism (3). Vitamin D is a fat-soluble vitamin that has several different metabolites in the human body. Two of the most important forms are 25-hydroxyvitamin D and 1,25-dihydroxyvitamin D, the storage and active form of Vitamin D, respectively (4). Recent studies have discovered that 1,25-dihydroxyvitamin D is consumed in the fracture region due to the reproduction of cells in that area. However, the molecular mechanism is not clearly understood (5). In addition, receptors for the active form of vitamin D appear on callus bone at the beginning of the recovery process (2). Various studies performed on animals, monitoring vitamin D level changes during the fracture healing process, reported a decreased level of vitamin D in the blood. Unfortunately, similar studies on human beings have shown mixed results, making it difficult to discern the truth about levels of vitamin D and its metabolites in serum during fractures healing (1). Considering the high prevalence of tibial and femoral fractures, and the fact that both fractures take similar times to heal, should lead to similar results with regards to vitamin D level changes during the healing process (3, 4, 6). 

## 2. Objectives

This study has been performed to obtain further information about the role of vitamin D supplementation during the process of bone formation and to relate changes in the level of various vitamin D metabolites to the process of fracture healing. If supplemental vitamin D is found to be beneficial, the administration of prophylactic therapy to prevent decreasing serum levels of vitamin D in patients with bone fractures may reduce complications. The aim of this study was to determine vitamin D serum level changes in the curative period of tibial and femoral shaft fractures in patients presented to the Poursina Hospital.

## 3. Patients and Methods

This cross-sectional study enrolled 73 patients with tibial and femoral shaft fractures referred to the Poursina Hospital, Rasht, IR Iran, between February 2011 and February 2012. All patients with tibial and femoral shaft fractures during their healing period of fracture were included after obtaining their informed consent. Exclusion criteria were other fracture and mild or severe vitamin D deficiency. Changes of vitamin D serum level were assessed three times in three weeks (at the first visit, end of the first week and end of the third week). Patients were informed about the study by phone and were invited to the hospital where blood samples were taken and sent to the Sina laboratory (Sina Darou Laboratories Company, Teheran, IR Iran). The serum level of 25-hydroxyvitamin D was evaluated with the radioimmunoassay method by commercial kits (DRG, Austria). The range of vitamin D was defined as: toxicity possible > 100 ng/mL, normal range 32 - 100 ng/mL, mild deficiency 15 - 32 ng/mL and severe deficiency < 15 ng/mL (7). Variables such as age, gender, fractured bone (tibia, femur or both), concomitant fracture of tibia and fibula, type of fracture (open or closed) and time of measurement, were also assessed. Statistical analyses were performed with SPSS software, version 18 (IBM, Armonk, NY, USA). Collected data were presented as frequency, mean ± SD and 95% confidence interval (95% CI). Data were analyzed using the paired t-test, the repeated measure analysis by epsilon test and the repeated measure ANOVA.

## 4. Results

**Figure 1.**
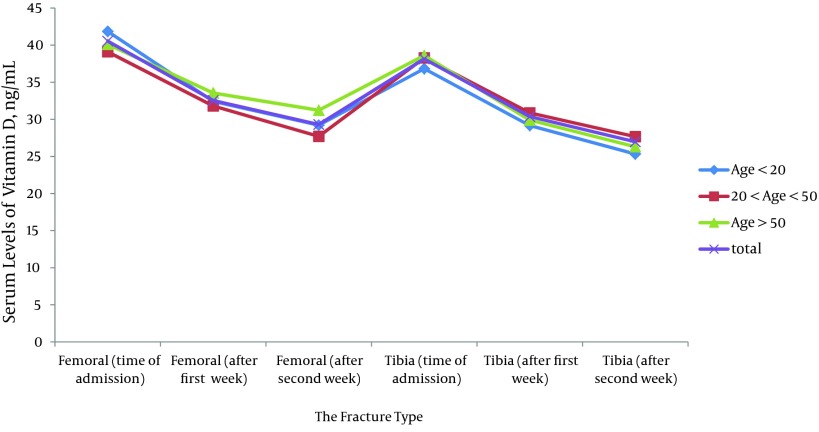
The Serum Levels of Vitamin D Divided into Age Groups

The serum levels of vitamin D were assessed in a total of 244 patients with a mean level of 18.92 ng/mL. A total of 171 patients (70%) were excluded because of vitamin D deficiency (mean level of 10.25 ng/mL). Finally, 73 patients aged between 1 and 87 years, with mean levels of vitamin D, and a mean age of 38.43 ± 22.00 years, continued the study; 57 patients (78.1%) were male and 16 patients (21.9%) were female. Forty tibia fractures and 33 femoral fractures were recorded. Also, an open fracture was present in 13 patients (17.8%). In 40 patients with fracture of tibia, 17 patients had concomitant fracture of the fibula. Furthermore, no significant change in vitamin D plasma levels was seen between patients with concomitant fracture of the tibia and fibula bones and patients with isolated fractures of the tibia (P < 0.353). In the 73 patients, the mean serum levels of vitamin D at time of admission, after one week and at the end of the third week were 39.23, 31.49 and 28.57 ng/mL, respectively. The mean serum level of vitamin D in patients with fracture of tibia or femur at first visit, were 40.54 and 38.15 ng/mL, respectively (Table 1). In patients younger than 6 years-old (six patients), the serum levels of vitamin D were reduced in the first week but increased during the second week (Figure 1). 

According to Table 2, the total serum levels of vitamin D in patients with fracture of tibia or femur were significantly reduced during curative period of fracture, (*P < 0.001 and P* < 0.0001, respectively). The overall reduction of vitamin D level was significantly more pronounced in the first week compared to the other weeks (P < 0.0001). The reduction of vitamin D levels was similar in tibia and femur fracture (P < 0.181). The changes in plasma levels of vitamin D were similar between genders in patients with fracture of tibia (P < 0.186) and femur (P < 0.686). There were no significant differences between age groups in fracture of the tibia (P < 0.941) and femur (P < 0.541). In addition, no significant changes of vitamin D levels were seen in different types of fractures between the tibia and the femur (P < 0.651 and P < 0.7, respectively). 

**Table 1.** Mean Serum Level of Vitamin D at the First Visit, End of the First Week and End of the Third Week

Serum Level of Vitamin D	Male, ng/mL	Female, ng/mL	Total, ng/mL
**Femur**			
Time of admission	41.86	37.50	40.54
After one week	32.43	32.80	32.54
At the end of the third week	28.86	30.30	29.30
**Tibia**			
Time of admission	39.02	33.16	38.15
After one week	31.41	26.16	30.26
At the end of the third week	27.97	28.00	27.97

**Table 2.** Reduction of Vitamin D Level in the First Week *vs.* the First Month

Fracture	Reduction of Vitamin D Level in the First Week *vs.* the First Month		P value
	Mean ± SD, ng/mL	95% Confidence Interval	
**Femur**	4.75 ± 3.96	[3.35 - 6.16]	0.0001
**Tibia**	4.87 ± 8.54	[2.13 - 7.61]	0.001
**Total**	4.82 ± 6.84	[3.22 - 6.41]	0.0001

## 5. Discussion

Vitamin D and its metabolites play an important role in bone metabolism and fracture repair (8, 9). Previous studies reporting decreases in serum vitamin D levels during the fracture healing process have been recorded for animals (3, 4). However, these same changes have not been well documented in the literature for humans. A high prevalence (43%) of low serum levels of vitamin D was reported by Bogunovic et al. (2) within the adult orthopedic surgery population, indirectly showing vitamin D deficiency in the acute post-fracture period. Several guidelines recommending at least 800 IU vitamin D per day for the primary prevention of low-trauma fractures among older individuals are in place (6, 8, 9). For falls and non-vertebral fracture prevention, the Swiss guidelines for the prevention of osteoporosis recommend 800 IU vitamin D for older individuals with limited sun exposure (4, 10). This recommendation was based on a quasi-consensus of vitamin D experts reviewing the evidence on bone health, fracture incidence and fall prevention in the elderly. In a recent study, Fu et al. (11) provided evidence that 1,25-dihydroxyvitamin D3 administrated by oral gavage could promote fracture healing in oophorectomized rats by increasing mechanical strength and improving the microstructure of the bony callus. In our study, 70% of patients were excluded because of vitamin D deficiency. In a study by Hovsepian et al. (12), 70.4% of adult patients were found to be vitamin D deficient in a population sampled around central Iran. Kaykhaei et al. (8) reported inadequate vitamin D status in 94.7% of patients the southeast region of Iran. Another study in Iran reported that 84% of students have vitamin D deficiency. The high prevalence of vitamin D deficiency found in Iran may be due to culture specific behaviors, beliefs, and regulations leading to decreases in the surface area of skin exposed to the sun. In our study, the mean vitamin D level at time of admission was 39.23 ng/mL. The reduction of vitamin D level was significantly more prominent in the first week versus the following weeks. Jingushi et al. (4) assessing the accumulation of serum 1a,25-dihydroxyvitamin D3 into the fracture callus during rat femoral fracture healing, demonstrated a dramatic fall in the plasma concentration of 1,25-dihydroxyvitamin D3occurring within 3 days from the fracture and persisting up to 10 days. Lidor et al. (6) showed that the levels of 3H-24,25-dihydroxyvitamin D3 were found to coincide with the formation of cartilaginous tissue in chicks with fractures. Their data also indicated that during the healing process, the plasma levels of 3H-1,25-dihydroxyvitamin D3 were below normal, while concentrations of 3H-1,25-dihydroxyvitamin D3 increased in the callus, diaphysis, and epiphysis on days 7 - 11 after fracture, compared with the control chicks. Current studies have shown that this hormone stimulates chondrogenesis by cell proliferation and promotion of matrix protein synthesis (12-14). Additionally 1,25-dihydroxyvitamin D3 acts directly on osteoblasts, stimulating the synthesis of osteocalcin (15-17) while also acting on osteoclasts to stimulate bone resorption (17-19). In a study by Lidor et al. (6), a significant increase in levels of the active metabolite of vitamin D were found in the callus during the first few days after fractures in chicks, and then again observed a decrease by the end of the third week. Meller et al. (5) found that there was a decrease in serum levels of 1,25-dihydroxyvitamin D3 on admission and up to 8 weeks later. In another study by Meller et al. (7), a significant reduction in serum levels of 25-hydroxyvitamin D along with concomitant rises in serum levels of PTH, 24,25-dihydroxyvitamin D and alkaline phosphatase were noted. It seems that consumption in the first week after fracture, due to the intense activity of osteoblasts and the higher speed of bone formation, is more remarkable compared to the other weeks measured in our study. An important difference between the previously mentioned studies and our study is that the previous studies were performed on rats (1-3, 5-7), while our study is the first study to be performed on humans. Our study shows that serum levels of vitamin D in patients with fractures of the tibia or femur are significantly reduced. In conclusion, serum levels of vitamin D in patients with fractures of the tibia or femur are reduced during the curative period of the fracture. This can be related to the role of vitamin D in the formation and mineralization of callus bone. Patients with tibial or femoral shaft fractures in the acute post-fracture curative period may benefit from the administration of vitamin D supplements. In addition, it is suggested to evaluate the exact change and role of vitamin D in the process of bone healing, in the future.
